# Supplementary material for: Repressed Blautia-acetate immunological axis underlies breast cancer progression promoted by chronic stress
Source: Nat Commun. 2023 Oct 3;14:6160. doi: 10.1038/s41467-023-41817-2 (PMC10547687; doi:10.1038/s41467-023-41817-2)
Supplement: Supplementary file 1 — Supplementary Information [file 41467_2023_41817_MOESM1_ESM.pdf]

Repressed Blautia-acetate immunological axis underlies breast cancer progression promoted by chronic stress

Ling Ye[1, #], Yuanlong Hou[2, 3 #], Wanyu Hu[1, #], Hongmei Wang[4], Ruopeng Yang[1], Qihan Zhang[5], Qiaoli Feng[5], Xiao Zheng[2], Guangyu Yao[5, *], Haiping Hao[2, *]

[1]NMPA Key Laboratory for Research and Evaluation of Drug Metabolism, Guangdong Provincial Key Laboratory of New Drug Screening, School of Pharmaceutical Sciences, Southern Medical University, Guangzhou, 510515, China.

[2]State Key Laboratory of Natural Medicines, Jiangsu Province Key Laboratory of Drug Metabolism, China Pharmaceutical University, Nanjing, 210009, China.

[3]Department of Pharmacy, Shenzhen Luohu People's Hospital, Shenzhen, 518000, China.

[4]Department of Radiation Oncology, Nanfang Hospital, Southern Medical University, Guangzhou, 510515, China

[5]Breast Center, Department of General Surgery, Nanfang Hospital, Southern Medical University, Guangzhou, 510515, China.

[#] These authors contribute equally to this work.

* Correspondence: haipinghao@cpu.edu.cn (Haiping Hao)

yaogy@smu.edu.cn (Guangyu Yao)

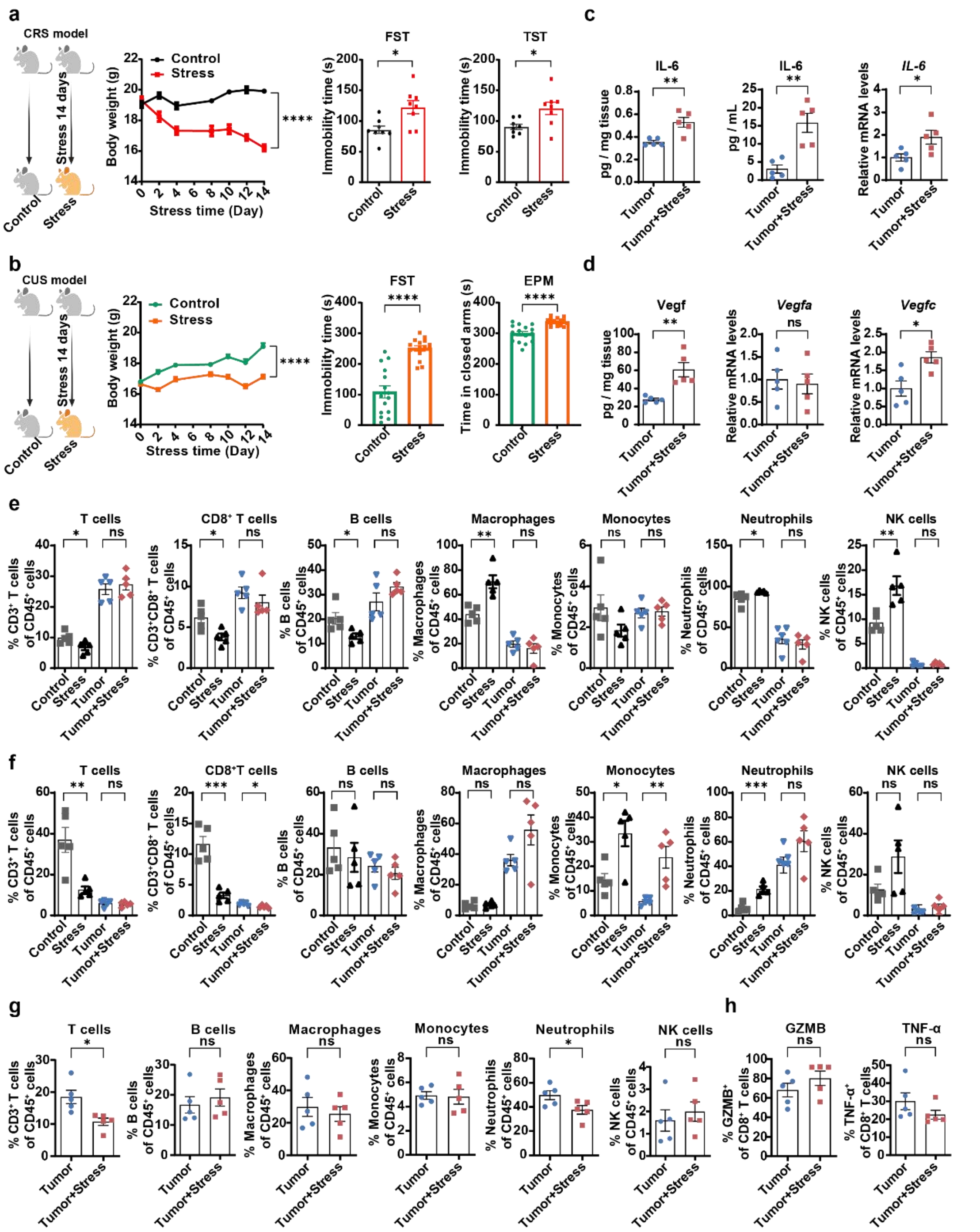

Supplementary Fig. 1. Chronic stress promotes breast cancer progression in mice. (a) Effects of chronic restraint stress (CRS) on body weight and immobility time of mice in the forced swim test (FST) and tail suspension test (TST), respectively (Body weight: n=11 per group, FST and TST: n=8 per group). Created with BioRender.com. (b) Effects of chronic unpredictable stress (CUS) on body weight and immobility time of mice in the FST and time in closed arms in elevated plus maze (EPM) test, respectively (Body weight: n=18 per group, FST and EPM: n=15 per group). Created

with BioRender.com. (c, d) Levels of IL-6 and Vegf in the serum and tumor tissue of CRS tumor mice (n=5 per group). (e, f) Abundance of immune cells in the blood and spleen of CRS mice (n=5 per group). (g) Tumor-infiltrated immune cells in CRS mice (n=5 per group). (h) Abundance of GZMB and TNF-α in tumor-infiltrated $CD8^+$ T cells in CRS mice (n=5 per group). Data were presented as mean ± SEM. Statistical analysis of body weight was determined using two-way ANOVA followed by Sidak's multiple comparison test and the other data were determined using unpaired two-tailed Student's t-test. **p* < 0.05; ***p* < 0.01; ****p* < 0.001; *****p* < 0.0001; ns, not significant. Source data and exact *p* values are provided in the Source data file.

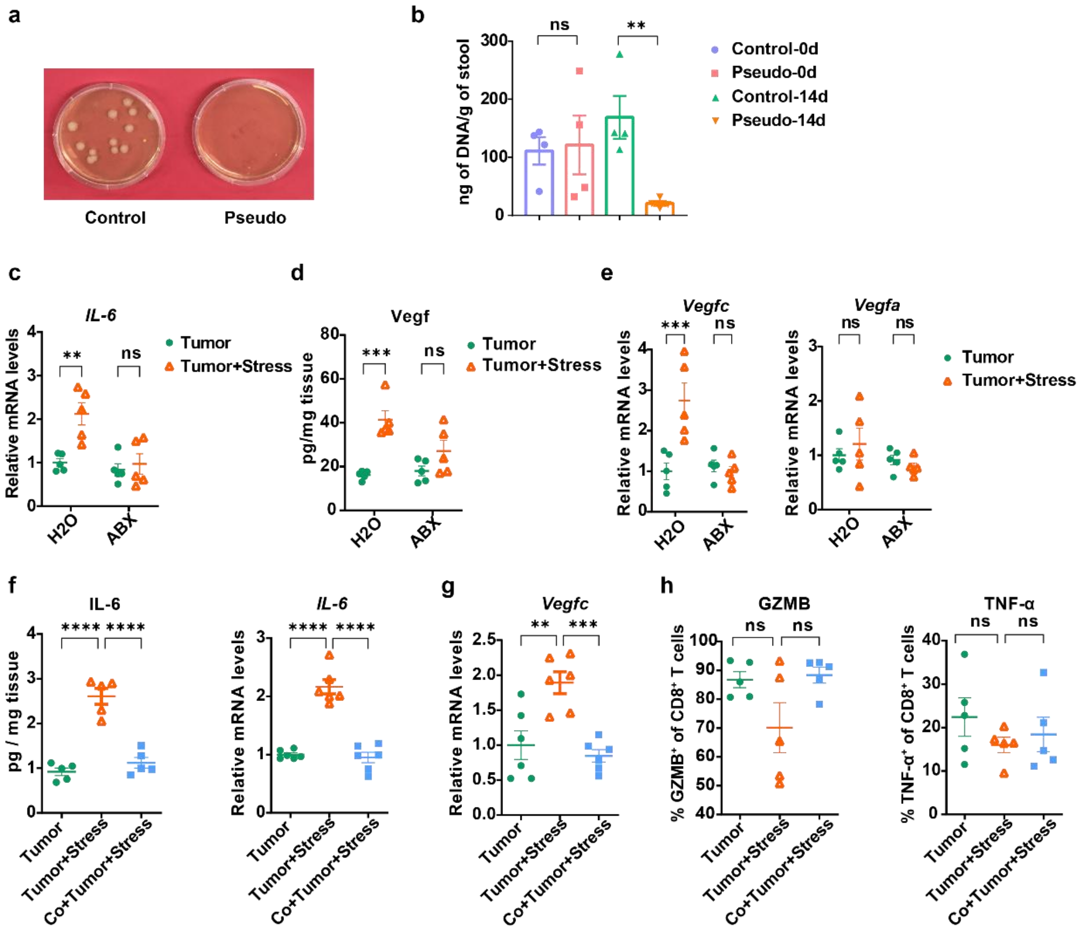

Supplementary Fig. 2. Chronic stress alters tumoral inflammatory cytokines involving gut microbiota. (a) Antibiotic-induced microbiome depletion illustrated through sample stool cultures of control and pseudo mice. (b) DNA extraction performed on stool samples from both control mice and pseudo mice (n=4 per group).

(c) Expression of *IL-6* in tumor tissue of non-stressed and stressed tumor groups with or without the antibiotic cocktail (ABX) treatment (n=5 per group). (d) Content of Vegf in the tumor tissue of non-stressed and stressed tumor groups with or without ABX treatment (n=5 per group). (e) Expression levels of *Vegfc* and *Vegfa* in the tumor tissue of non-stressed and stressed tumor groups with or without ABX treatment (n=5 per group). (f) Content (n=5 per group) and expression level (n=6 per group) of IL-6 in the tumor tissue of different groups in the co-housing experiments. (g) Expression level of *Vegfc* in the tumor tissue of different groups in the co-housing experiments (n=6 per group). (h) GZMB and TNF-α abundance of tumor-infiltrated $CD8^+$ T cells in the co-housing experiments (n=5 per group). Data were presented as mean ± SEM. Statistical significance was determined by (b) unpaired two-tailed Student's t-test, (c-e) two-way ANOVA followed by Sidak's multiple comparison test, or (f-h) one-way ANOVA followed by Tukey's multiple comparisons test. *$p < 0.05$; **$p < 0.01$; ***$p < 0.001$; ****$p < 0.0001$; ns, not significant. Source data and exact *p* values are provided in the Source data file.

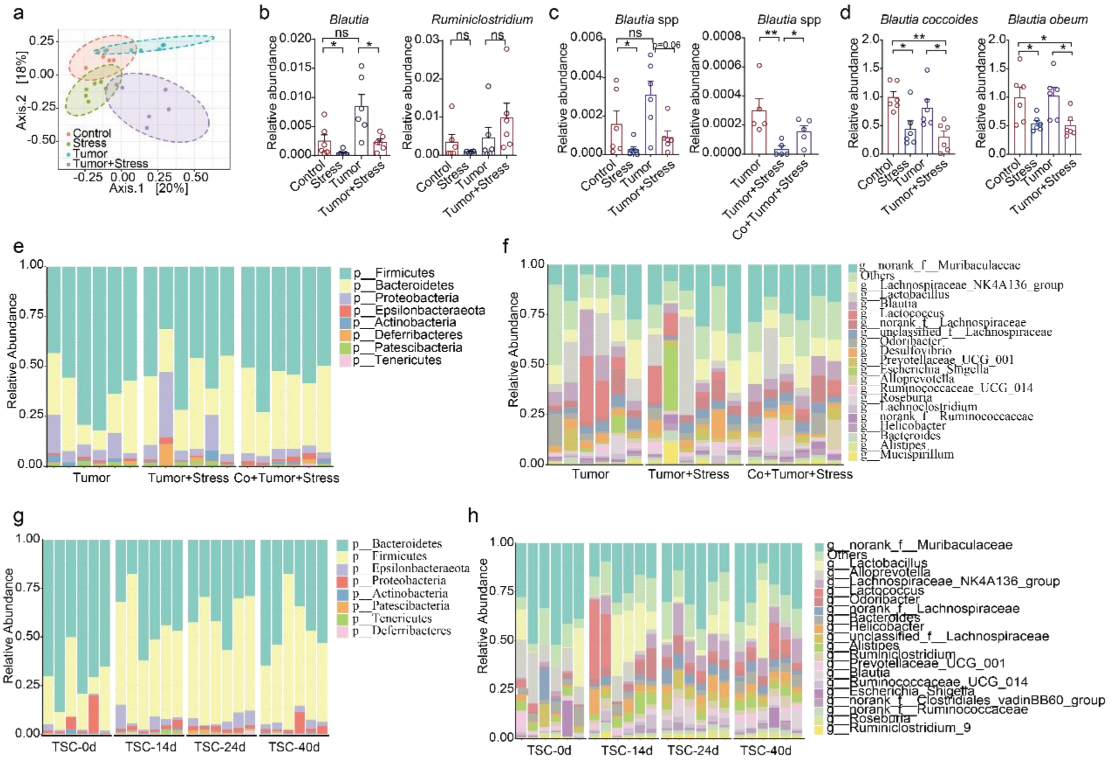

Supplementary Fig. 3. Chronic stress remodels the composition of the gut microbiota. (a) Principal Coordinate Analysis (PCoA) plot of the cecal microbial composition of non-stressed and stressed mice with or without breast cancer at the end of 40 days. The community distance is based on the Bray-Curtis dissimilarity metrics of relative abundance determined by 16S rRNA sequencing. Each symbol represents the data of an individual mouse (n=6 per group). (b) Relative abundance of *Blautia* and *Ruminiclostridium* at the genus level in the cecal sample by 16S rRNA sequencing (n=6 per group). (c) Relative abundance of *Blautia* spp in the cecal sample by 16S rRNA sequencing (Control, Stress, Tumor, Tumor+Stress group: n=6, Tumor, Tumor+Stress, Co+Tumor+Stress group: n=5). (d) Analysis of gene expression representative of *Blautia coccoides* and *Blautia obeum* in fecal samples from CUS mice by qPCR. (e, f) Average relative abundance of representative phylum (e) and genus (f) in the gut microbiota from the co-housing experiment by 16S rRNA sequencing (n=6 per group). (g, h) Time-dependent changes in relative abundance at the phylum (g) and genus (h) level from the fecal microbiota of co-housed mice by 16S rRNA sequencing (n=6 per group). Data were presented as mean ± SEM. *$p <$ 0.05 by the Wilcoxon rank-sum test. "ns" means no significant difference. Source data

and exact *p* values are provided in the Source data file.

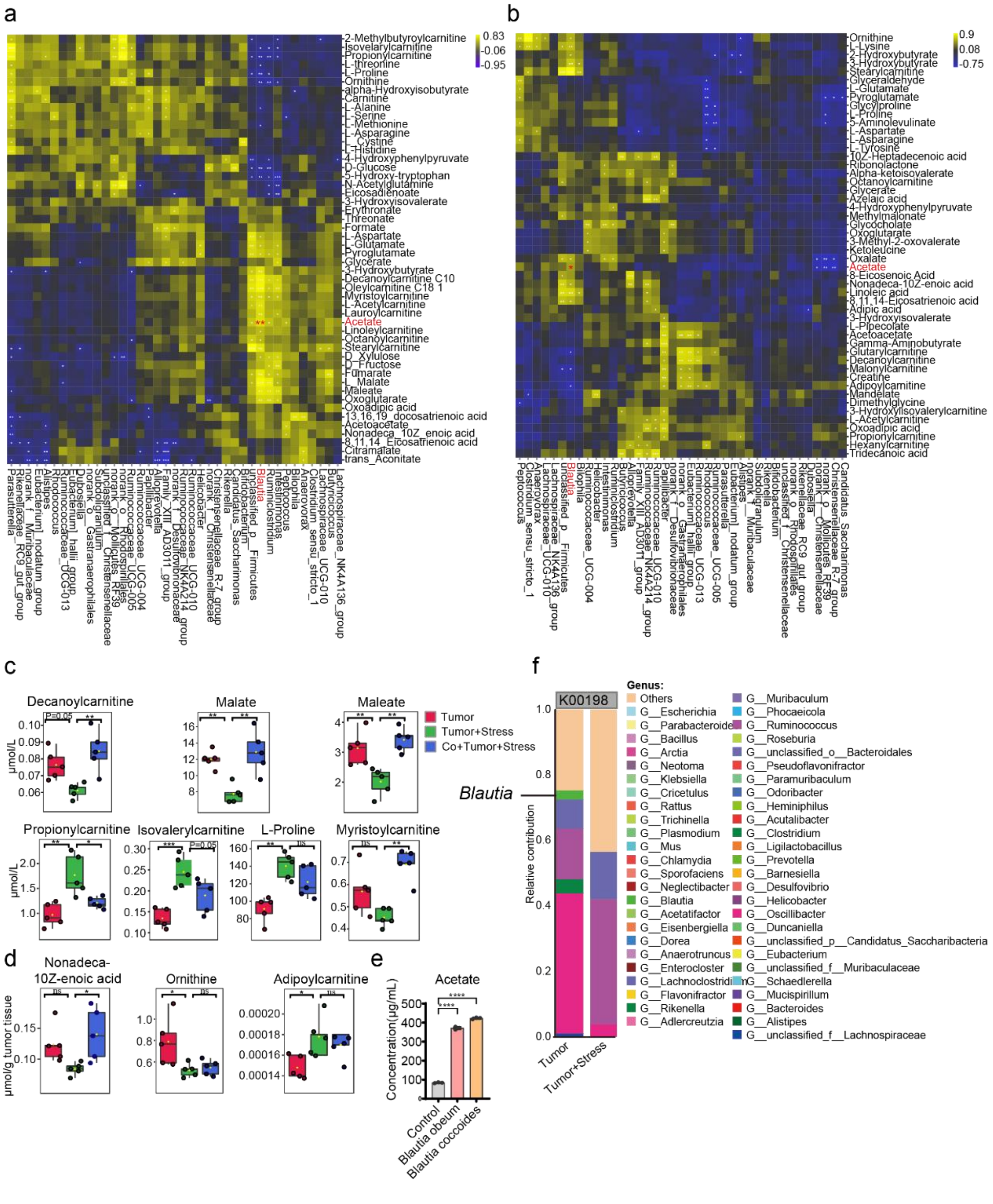

Supplementary Fig. 4. Altered profiles of endogenous metabolites and their correlations with microbial genus abundances. (a) Spearman correlation analysis between microbial genus abundances and serum metabolites (n=5 per group). Each spot in the heatmap represents the Spearman correlation coefficient (R value) between microbial abundance and the concentration of a specific serum metabolite. The color of each spot indicates the strength and direction of the correlation: yellow color

indicates significant positive correlation (R > 0.3, *$p < 0.05$; **$p < 0.01$; ***$p < 0.001$), whereas blue spot indicates significant negative correlation (R < −0.3 and *$p < 0.05$; **$p < 0.01$; ***$p < 0.001$). (b) Spearman correlation analysis between microbial genus abundances and tumor metabolites. The heatmap shows the R values of the Spearman correlation analysis between the abundance of microbial genera and the concentration of tumor metabolites. The color scheme follows the same interpretation as described for panel (a). Concentration of significant changes of other metabolites in the (c) serum and (d) tumor tissue (n=5 per group). The boxplot displays the distribution of concentrations for each metabolite, with the box representing the interquartile range (10th to 90th percentiles), the center line representing the median, and the whiskers representing the minimum and maximum values. (e) The concentration of acetate in the culture medium of *Blautia coccoides* and *Blautia obeum* (n=3 per group). (f) Barplot of species and functional contribution analysis, highlighting the top 50 genus contributing the K00198. Data were presented as mean ± SEM. Statistical significance was determined by the one-way ANOVA followed by the Holm-Sidak test for panels (c) and (d). *$p < 0.05$, **$p < 0.01$, ***$p < 0.001$, ****$p < 0.0001$; “ns” means no significant difference. Source data and exact p values are provided in the Source data file.

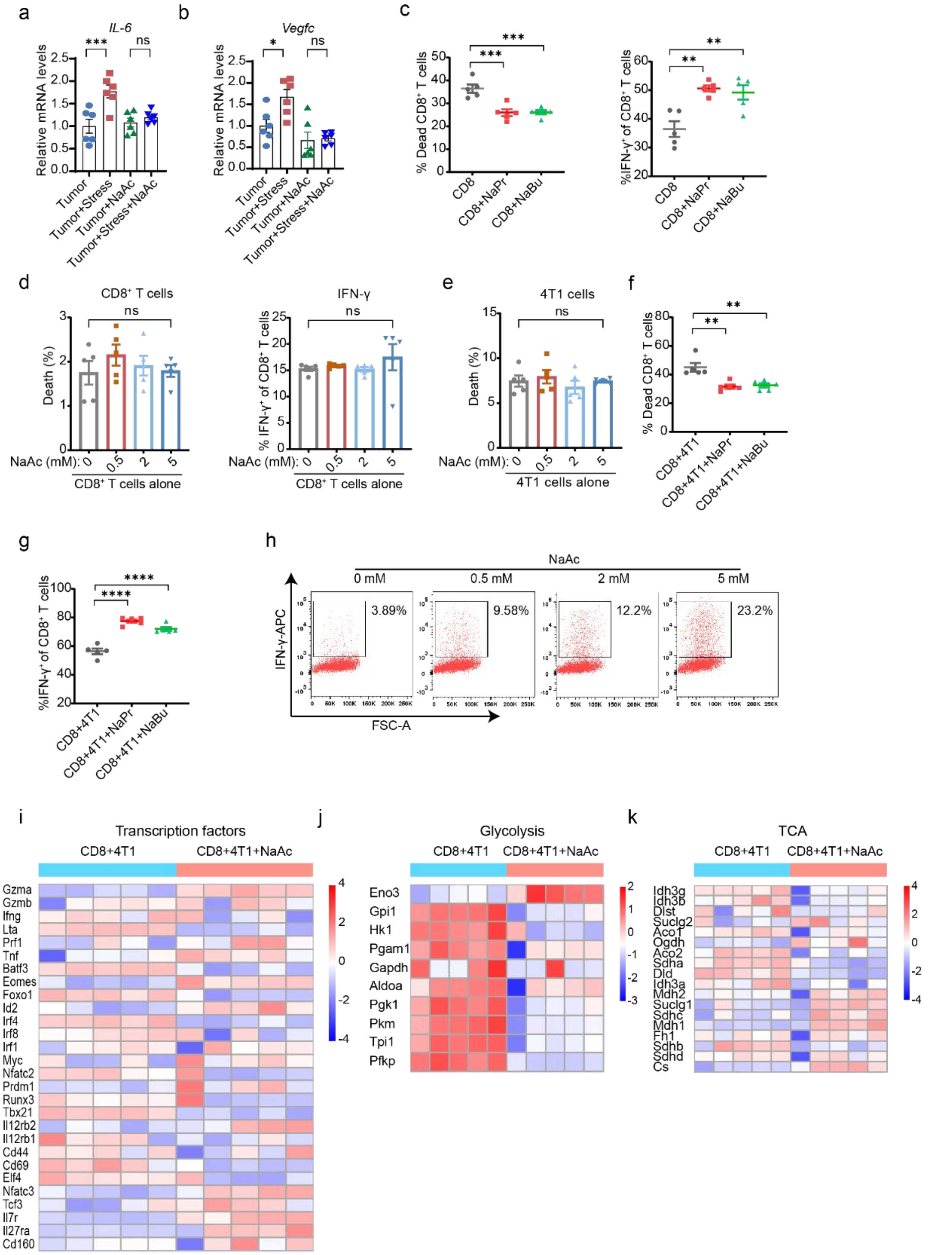

Supplementary Fig. 5. The effects of sodium acetate (NaAc), sodium propionate (NaPr) and sodium butyrate (NaBu) on the CD8$^+$ T cells. (a) Expression level of *IL-6* in the tumor tissue of different treatment groups (n=6 per group). (b) Expression level of *Vegfc* in the tumor tissue of different treatment groups (n=6 per group). (c) Percentage of CD8$^+$ T cell death after NaPr and NaBu treatment and IFN-γ abundance of CD8$^+$ T cells (n=5 per group). (d) Percentage of CD8$^+$ T cell death after NaAc

treatment and IFN-γ abundance of CD8$^+$ T cells (n=5 per group). (e) Percentage of 4T1 cancer cell death after NaAc treatment (n=5 per group). (f, g) Percentage of CD8$^+$ T cell death in co-culture with 4T1 cells after NaPr and NaBu treatment and IFN-γ abundance of CD8$^+$ T cells (n=5 per group). (h) Representative flow plots showing IFN-γ of CD8$^+$ T cells co-cultured with 4T1 cells in the presence or absence of NaAc treatment. Heatmaps for gene expression of transcription factors regulating CD8$^+$ T cell effector, memory and exhaustion (i), glycolysis (j) and TCA cycle (k) of control and acetate-exposed CD8$^+$ T cells during co-culture with 4T1 cells (n=5 per group). Data were presented as mean ± SEM. Statistical significance was determined by one-way ANOVA followed by Tukey's multiple comparisons test. *$p < 0.05$; **$p < 0.01$; ***$p < 0.001$; ****$p < 0.0001$. ns means no significant difference. Source data and exact p values are provided in the Source data file.

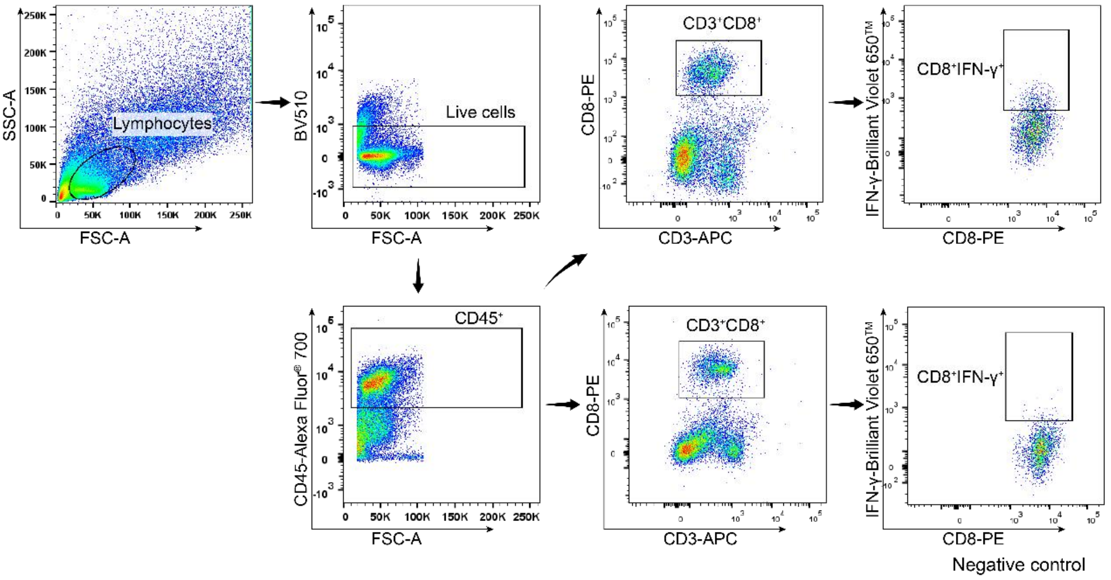

Supplementary Fig. 6. Gating strategy for the detection of IFN-γ of CD8$^+$ T cells in the tumor tissue by flow cytometry.

Supplementary Table 1. The primer sequences used for PCR reaction.

Gene name	Forward primer (5’-3’)	Reverse primer (5’-3’)
IL-6	CAACGATGATGCACTTGCAGA	TGTGACTCCAGCTTATCTCTTGG
Tnfa	CTCAGCGAGGACAGCAAGG	AGGGACAGAACCTGCCTGG
Vegfa	AATGCTTTCTCCGCTCTGAA	GCTTCCTACAGCACAGCAGA
Vegfc	GAGGTCAAGGCTTTTGAAGGC	CTGTCCTGGTATTGAGGGTGG
IFN-γ	GTTACTGCCACGGCACAGTCATTG	ACCATCCTTTTGCCAGTTCCTCCAG
Gapdh	TGAAGCAGGCATCTGAGGG	CGAAGGTGGAAGAGTGGGAG
Blautia coccoides	CGGTACCTGACTAAGAAGC	AGTTTCATTCTTGCGAACG
Blautia obeum	TGGGTGTAAAGGGAGCGTAG	CTTTCGAGCCTCAACGTCAG
16S rRNA	ACTCCTACGGGAGGCAGCAG	ATTACCGCGGCTGCTGG
Acly	ACCCTTTCACTGGGGATCACA	GACAGGGATCAGGATTTCCTTG
Acss1	GTTTGGGACACTCCTTACCATAC	AGGCAGTTGACAGACACATTC
Acss2	GTGAAAGGATCTTGGATTCCAGT	CAGATGTTTGACCACAATGCAG
Slc16a1	GGCAGCCGTCCAGTAATGAT	TGAAAGCAAGCCCAAGACCT
Slc16a3	GGCGGTAACAGGTGAAAGCA	GCGTAGGAGAAACCCGTGAT
18S rRNA	CGGCGACGACCCATTCGAAC	GAATCGAACCCTGATTCCCCGT

Supplementary Table 2.

Reagent	Source	Dilution	Identifier
Antibodies			
Trustain FCX™-anti-mouse CD16/32	BioLegend	1/200	Clone: 93; Cat#101319; RRID: AB_1574973
Brilliant Violet 510™-anti-mouse CD3	BioLegend	1/100	Clone: 17A2; Cat#100233; RRID: AB_2561387
APC anti-mouse CD3	BioLegend	1/100	Clone: 17A2; Cat#100235; RRID: AB_2561455
Brilliant Violet 421™ anti-mouse CD3ε	BioLegend	1/100	Clone: 145-2C11; Cat#100335; RRID: AB_10898314
FITC anti-mouse CD3	BioLegend	1/100	Clone: 17A2; Cat#100203; RRID: AB_312660
Alexa Fluor® 700-anti-mouse CD45	BioLegend	1/200	Clone: 30-F11; Cat#103127; RRID: AB_493714
FITC-anti-mouse CD4	BioLegend	1/200	Clone: GK1.5; Cat#100405; RRID: AB_312690

PerCP-cyanine5.5-anti-mouse CD8a	BioLegend	1/250	Clone: 53-6.7; Cat#100733; RRID: AB_2075239
PE-anti-mouse CD8a	BioLegend	1/250	Clone: 53-6.7; Cat#100707; RRID: AB_312746
FITC anti-mouse CD8a	BioLegend	1/250	Clone: 53-6.7; Cat#100706; RRID: AB_312745
FITC-anti-mouse CD19	BioLegend	1/200	Clone: 6D5; Cat#115505; RRID: AB_313640
APC-anti-mouse CD11b	BioLegend	1/100	Clone: M1/70; Cat#101211; RRID: AB_312794
PerCp/Cyanine5.5 anti-mouse/human CD11b	BioLegend	1/100	Clone: M1/70; Cat#101227; RRID: AB_893233
PE-Cyanine^{7}-anti-mouse CD11c	BioLegend	1/100	Clone: N418; Cat#117317; RRID: AB_493569
Brilliant Violet 421™-anti-mouse I-A/I-E	BioLegend	1/100	Clone M5/114.15.2; Cat# 107632; RRID: AB_2650896
Brilliant Violet 605™-anti-mouse Ly-6C	BioLegend	1/100	Clone: HK1.4; Cat#128036; RRID: AB_2562353
PerCP-cyanine5.5-anti-mouse Ly-6G	BioLegend	1/200	Clone: 1A8; Cat#127615; RRID: AB_1877272
PE-anti-mouse Ly-6G	BioLegend	1/200	Clone: 1A8; Cat#127607; RRID: AB_1186104
APC-anti-mouse CD49b (Pan-NK)	BioLegend	1/500	Clone: DX5; Cat#108909; RRID: AB_313416
Brilliant Violet 650™-anti-mouse IFN-γ	BioLegend	1/100	Clone: XMG1.2; Cat#505832; RRID: AB_2734492
APC-anti-mouse IFN-γ	BioLegend	1/100	Clone: XMG1.2; Cat#505810; RRID: AB_315404
PE/Cyanine7-anti-mouse TNF-α	BioLegend	1/100	Clone: MP6-XT22; Cat#506323; RRID: AB_2204356
FITC-anti-mouse Granzyme B	BioLegend	1/100	Clone: GB11; Cat#515403; RRID: AB_2114575
Anti-Mouse CD3 SAFIRE Purified	Peprotech/Biogems	1/1000	Clone: 17A2; Cat#05112-25-500
Anti-Mouse CD28 SAFIRE Purified	Peprotech/Biogems	1/2000	Clone: 37.51; Cat#10312-25-500
In Vivo Mab anti-mouse CD8α	Bioxcell	1/6	Clone: 2.43; Cat#BE0061
Anti-CD8 antibody	ZSGB-Bio	N/A	Clone: SP16; Cat#ZA-0508
Chemicals			
Annexin V-FITC/PI KIT	ES Science		Cat# AP001-100
7-AAD Viability Staining Solution	BioLegend		Cat#420403
Zombie Aqua™ Fixable Viability Kit (DMSO)	BioLegend		Cat#423101
Fixation Buffer	BioLegend		Cat#420801
Perm Buffer Set	BioLegend		Cat#421403
Intracellular Staining Permeabilization Wash Buffer	BioLegend		Cat#421002
Brefeldin A Solution	BioLegend		Cat#420601

Cell Activation Cocktail (without Brefeldin A)	BioLegend	Cat#423301
Bouin's Fixative Solution	Phygene	Cat#PH0976
Mouse IL-6 ELISA Kit	ExCell Bio	Cat#EM004-96
Mouse VEGF ELISA Kit	ExCell Bio	Cat#EM009-96
Mouse IFN-γ ELISA Kit	R&D Systems	Cat#MIF00
Mouse acetyl-CoA ELISA Kit	CUSABIO	Cat#CSB-E12936m
Transcription Factor Buffer Set 100 Tst	BD Pharmingen	Cat#562574
Easy Sep^{TM} Mouse CD8^{+} T cell isolation Kit	Stemcell	Cat#19853
Easy Sep^{TM} Buffer	Stemcell	Cat#20144
Murine IL-2	Peprotech	Cat#212-12-20
Sodium acetate	Aladdin	Cat#S118649
Sodium propionate	Aladdin	Cat#S100121
Sodium butyrate	Sigma	Cat#V900464
Fetal Bovine Serum (FBS)	Gibco	Cat#10270-106
RPMI 1640 Medium	Gibco	Cat#11875085
RPMI 1640 Medium, no glucose	Gibco	Cat#11879020
Penicillin-Streptomycin	Gibco	Cat#15140-122
Collagenase II	Sigma-Aldrich	Cat#C6885
Collagenase IV	Sigma-Aldrich	Cat#C5138
DNase I	Sigma-Aldrich	Cat#DN25
Ampicillin sodium salt	Aladdin	Cat#A105483
Neomycin sulfate	Aladdin	Cat#N109017
Metronidazole	Aladdin	Cat#M109874
Vancomycin	Aladdin	Cat#V301569
Experimental Models: Cell Lines/Bacterial Strains		
Murine breast cancer 4T1	ATCC	Cat#CRL-2539
E0771	ATCC	Cat#CRL-3461
Blautia coccoides	DSMZ	Cat#DSM 26115
Blautia obeum	DSMZ	Cat#DSM 25238

Experimental Models: Organisms/Strains		
Mouse: Female BALB/c mice (6-week-old)	Beijing Vital River Laboratory Animal Technology	N/A
Mouse: Female C57BL/6 mice (6-week-old)	Beijing Vital River Laboratory Animal Technology	N/A
Software and Algorithms		
GraphPad Prim 7.0	GraphPad Software	https://www.graphpad.com
FlowJo version 10	FlowJo	https://www.flowjo.com
