# Supplementary material for: Repressed Blautia-acetate immunological axis underlies breast cancer progression promoted by chronic stress
Source: Nat Commun. 2023 Oct 3;14:6160. doi: 10.1038/s41467-023-41817-2 (PMC10547687; doi:10.1038/s41467-023-41817-2)
Supplement: Supplementary file 3 — Reporting Summary [file 41467_2023_41817_MOESM3_ESM.pdf]

nature portfolio

Corresponding author(s): Haiping Hao

Last updated by author(s): Sep 5, 2023

Reporting Summary

Nature Portfolio wishes to improve the reproducibility of the work that we publish. This form provides structure for consistency and transparency in reporting. For further information on Nature Portfolio policies, see our Editorial Policies and the Editorial Policy Checklist.

Statistics

For all statistical analyses, confirm that the following items are present in the figure legend, table legend, main text, or Methods section.

n/a	Confirmed	
☐	☒	The exact sample size (n) for each experimental group/condition, given as a discrete number and unit of measurement
☐	☒	A statement on whether measurements were taken from distinct samples or whether the same sample was measured repeatedly
☐	☒	The statistical test(s) used AND whether they are one- or two-sided<br>*Only common tests should be described solely by name; describe more complex techniques in the Methods section.*
☒	☐	A description of all covariates tested
☐	☒	A description of any assumptions or corrections, such as tests of normality and adjustment for multiple comparisons
☐	☒	A full description of the statistical parameters including central tendency (e.g. means) or other basic estimates (e.g. regression coefficient) AND variation (e.g. standard deviation) or associated estimates of uncertainty (e.g. confidence intervals)
☒	☐	For null hypothesis testing, the test statistic (e.g. F, t, r) with confidence intervals, effect sizes, degrees of freedom and P value noted<br>*Give P values as exact values whenever suitable.*
☒	☐	For Bayesian analysis, information on the choice of priors and Markov chain Monte Carlo settings
☒	☐	For hierarchical and complex designs, identification of the appropriate level for tests and full reporting of outcomes
☐	☒	Estimates of effect sizes (e.g. Cohen's d, Pearson's r), indicating how they were calculated

Our web collection on statistics for biologists contains articles on many of the points above.

Software and code

Policy information about availability of computer code

Data collection	The targeted raw data were processed using the TMBQ software (v1.0, HMI, Shenzhen, Guangdong, China) to perform peak integration, calibration, and quantification for each metabolite. The current TMBQ (GNU GPL.V3 license) is implemented with the Java and R and freely available via http://119.136.25.134:9011. Flow cytometry were performed using LSR-Fortessa instruments (BD. Biosciences).
Data analysis	The raw data for the 16S rRNA were analyzed with QIME(version 4.2;http://drive5.com/uchime/uchime_download.html). Statistical analysis was performed using GraphPad Prism 7.0 (GraphPad Software, Inc., San Diego,CA,USA). Principal co--ordinates analysis (PCoA), non-metric multidimensional scaling (NMDS), and Lefse analysis were conducted using Microbiome Analyst (http://www.microbiomeanalyst.ca/).

For manuscripts utilizing custom algorithms or software that are central to the research but not yet described in published literature, software must be made available to editors and reviewers. We strongly encourage code deposition in a community repository (e.g. GitHub). See the Nature Portfolio guidelines for submitting code & software for further information.

Data

Policy information about availability of data

All manuscripts must include a data availability statement. This statement should provide the following information, where applicable:

- Accession codes, unique identifiers, or web links for publicly available datasets
- A description of any restrictions on data availability
- For clinical datasets or third party data, please ensure that the statement adheres to our policy

The 16S rRNA and metagenomic sequencing datasets are available in the NCBI SRA database under the accession numbers PRJNA912999 and PRJNA978792,

respectively [https://www.ncbi.nlm.nih.gov/bioproject/PRJNA912999; https://www.ncbi.nlm.nih.gov/bioproject/PRJNA978792]. Additionally, the RNA sequencing data have been deposited in GEO with the subseries code GSE231961 [https://www.ncbi.nlm.nih.gov/geo/query/acc.cgi?acc=GSE231961]. Source data are provided with this paper.

Human research participants

Policy information about studies involving human research participants and Sex and Gender in Research.

Reporting on sex and gender	We enrolled 52 female patients with breast cancer, out of which 24 were also diagnosed with depression. Given that breast cancer primarily affects females, our study exclusively included female participants.<br>In addition, we recruited 40 patients with major depressive disorder, comprising 12 males and 28 females. We also had 30 healthy donors, consisting of 7 males and 23 females. We did not perform sex- and gender-based analyses. In this study, we excluded sex as a variable in both the study design and analysis, as our primary focus was to investigate whether reduced Blautia-acetate levels observed in depressed mice could be replicated in depressed patients. Since sex was not a targeted variable in our study, and data pertaining to sex-specific differences were neither collected nor analyzed separately.
Population characteristics	No significant covariate factors, such as age, sex, past diagnoses, or treatments, were identified in this study.
Recruitment	Participants were recruited according to the study protocol for each project. Notably, there was no evident self-selection bias identified in this study.
Ethics oversight	Human stool samples were collected from both healthy controls and depression patients following ethical guidelines and with the approval of the Institutional Review Board of Southeast University Affiliated Zhongda Hospital (Reference 2018ZDSYLL119-P01). Human blood and breast tissue samples were collected with the approval of the Institutional Review Board of Nanfang Hospital (Reference NFEC-2018-038).

Note that full information on the approval of the study protocol must also be provided in the manuscript.

Field-specific reporting

Please select the one below that is the best fit for your research. If you are not sure, read the appropriate sections before making your selection.

[x] Life sciences   [ ] Behavioural & social sciences   [ ] Ecological, evolutionary & environmental sciences

For a reference copy of the document with all sections, see nature.com/documents/nr-reporting-summary-flat.pdf

Life sciences study design

All studies must disclose on these points even when the disclosure is negative.

Sample size	For our in vivo studies, we determined the sample size based on our extensive experience with animal models and endpoints. We selected appropriate sample sizes, as outlined in the manuscript, to ensure the generation of reproducible results with a significance level of less than 0.05 and a power of over 90%.<br>In the case of in vitro experiments, we chose sample sizes, with a minimum of three independent experimental replicates, in accordance with the standard practices of the research field (N=3). This selection was made to produce reproducible results with a significance level of less than 0.05 and a power exceeding 90%.<br>Regarding human samples, the total sample size was contingent upon the availability of samples and the characteristics of the patients.
Data exclusions	No data were excluded from the analyses.
Replication	We repeated experiments involving chronic stress and tumor induction in mice 2-3 times, consistently achieving satisfactory reproducibility. In vitro experiments conducted with 4T1 cells and/or CD8+ T cells were successfully replicated at least twice.For human research participants, we denoted the experimental points as 'n'-values, serving as biological replicates, in each figure legend. We utilized biological replicates for our statistical analyses, and we confirmed that all replication attempts in this study were successful.
Randomization	Allocation was random. Detailed definitions and descriptions were provided in the manuscript.
Blinding	Investigators were blinded to group allocation during data collection and analysis wherever possible. This was not possible during real-time treatment of live animals in mouse studies, as the treatment of each mouse would need to be known to the person handling the mice.

Behavioural & social sciences study design

All studies must disclose on these points even when the disclosure is negative.

Study description	*Briefly describe the study type including whether data are quantitative, qualitative, or mixed-methods (e.g. qualitative cross-sectional, quantitative experimental, mixed-methods case study).*
Research sample	*State the research sample (e.g. Harvard university undergraduates, villagers in rural India) and provide relevant demographic information (e.g. age, sex) and indicate whether the sample is representative. Provide a rationale for the study sample chosen. For studies involving existing datasets, please describe the dataset and source.*
Sampling strategy	*Describe the sampling procedure (e.g. random, snowball, stratified, convenience). Describe the statistical methods that were used to predetermine sample size OR if no sample-size calculation was performed, describe how sample sizes were chosen and provide a rationale for why these sample sizes are sufficient. For qualitative data, please indicate whether data saturation was considered, and what criteria were used to decide that no further sampling was needed.*
Data collection	*Provide details about the data collection procedure, including the instruments or devices used to record the data (e.g. pen and paper, computer, eye tracker, video or audio equipment) whether anyone was present besides the participant(s) and the researcher, and whether the researcher was blind to experimental condition and/or the study hypothesis during data collection.*
Timing	*Indicate the start and stop dates of data collection. If there is a gap between collection periods, state the dates for each sample cohort.*
Data exclusions	*If no data were excluded from the analyses, state so OR if data were excluded, provide the exact number of exclusions and the rationale behind them, indicating whether exclusion criteria were pre-established.*
Non-participation	*State how many participants dropped out/declined participation and the reason(s) given OR provide response rate OR state that no participants dropped out/declined participation.*
Randomization	*If participants were not allocated into experimental groups, state so OR describe how participants were allocated to groups, and if allocation was not random, describe how covariates were controlled.*

Ecological, evolutionary & environmental sciences study design

All studies must disclose on these points even when the disclosure is negative.

Study description	*Briefly describe the study. For quantitative data include treatment factors and interactions, design structure (e.g. factorial, nested, hierarchical), nature and number of experimental units and replicates.*
Research sample	*Describe the research sample (e.g. a group of tagged Passer domesticus, all Stenocereus thurberi within Organ Pipe Cactus National Monument), and provide a rationale for the sample choice. When relevant, describe the organism taxa, source, sex, age range and any manipulations. State what population the sample is meant to represent when applicable. For studies involving existing datasets, describe the data and its source.*
Sampling strategy	*Note the sampling procedure. Describe the statistical methods that were used to predetermine sample size OR if no sample-size calculation was performed, describe how sample sizes were chosen and provide a rationale for why these sample sizes are sufficient.*
Data collection	*Describe the data collection procedure, including who recorded the data and how.*
Timing and spatial scale	*Indicate the start and stop dates of data collection, noting the frequency and periodicity of sampling and providing a rationale for these choices. If there is a gap between collection periods, state the dates for each sample cohort. Specify the spatial scale from which the data are taken*
Data exclusions	*If no data were excluded from the analyses, state so OR if data were excluded, describe the exclusions and the rationale behind them, indicating whether exclusion criteria were pre-established.*
Reproducibility	*Describe the measures taken to verify the reproducibility of experimental findings. For each experiment, note whether any attempts to repeat the experiment failed OR state that all attempts to repeat the experiment were successful.*
Randomization	*Describe how samples/organisms/participants were allocated into groups. If allocation was not random, describe how covariates were controlled. If this is not relevant to your study, explain why.*
Blinding	*Describe the extent of blinding used during data acquisition and analysis. If blinding was not possible, describe why OR explain why blinding was not relevant to your study.*

Did the study involve field work? ☐ Yes ☐ No

Field work, collection and transport

Field conditions	*Describe the study conditions for field work, providing relevant parameters (e.g. temperature, rainfall).*
Location	*State the location of the sampling or experiment, providing relevant parameters (e.g. latitude and longitude, elevation, water depth).*
Access & import/export	*Describe the efforts you have made to access habitats and to collect and import/export your samples in a responsible manner and in compliance with local, national and international laws, noting any permits that were obtained (give the name of the issuing authority, the date of issue, and any identifying information).*
Disturbance	*Describe any disturbance caused by the study and how it was minimized.*

Reporting for specific materials, systems and methods

We require information from authors about some types of materials, experimental systems and methods used in many studies. Here, indicate whether each material, system or method listed is relevant to your study. If you are not sure if a list item applies to your research, read the appropriate section before selecting a response.

Materials & experimental systems

n/a	Involved in the study	
☐	☒	Antibodies
☐	☒	Eukaryotic cell lines
☒	☐	Palaeontology and archaeology
☐	☒	Animals and other organisms
☐	☒	Clinical data
☒	☐	Dual use research of concern

Methods

n/a	Involved in the study	
☒	☐	ChIP-seq
☐	☒	Flow cytometry
☒	☐	MRI-based neuroimaging

Antibodies

Antibodies used

1. Trustain FCXTM-anti-mouse CD16/32(Clone: 93; Cat#101319; RRID: AB_1574973, BioLegend),
2. Brilliant Violet 510TM-anti-mouse CD3(Clone: 17A2; Cat#100233; RRID: AB_2561387, BioLegend)
3.APC anti-mouse CD3(Clone: 17A2; Cat#100235; RRID: AB_2561455, BioLegend)
4. Brilliant Violet 421™ anti-mouse CD3ε(Clone: 145-2C11; Cat#100335; RRID: AB_10898314, BioLegend)
5. FITC anti-mouse CD3 (Clone: 17A2; Cat#100203; RRID: AB_312660, BioLegend)
6. Alexa 4.Fluor® 700-anti-mouse CD45(Clone: 30-F11; Cat#103127; RRID: AB_493714, BioLegend)
7. FITC-anti-mouse CD4(Clone: GK1.5; Cat#100405; RRID: AB_312690, BioLegend)
8.PerCP-cyanine5.5-anti-mouse CD8a(Clone: 53-6.7; Cat#100733; RRID: AB_2075239 , BioLegend)
9.PE anti-mouse CD8a(Clone: 53-6.7; Cat#100707; RRID: AB_312746, BioLegend)
10.FITC anti-mouse CD8a (Clone: 53-6.7; Cat#100706; RRID: AB_312745, BioLegend)
11.FITC-anti-mouse CD19(Clone: 6D5; Cat#115505; RRID: AB_313640, BioLegend)
12.APC-anti-mouseCD11b(Clone: M1/70; Cat#101211; RRID: AB_312794, BioLegend)
13. PerCp/Cyanine5.5 anti-mouse/human CD11b(Clone: M1/70; Cat#101227; RRID: AB_893233, BioLegend)
14. PE-Cyanine7-anti-mouse CD11c(Clone: N418; Cat#117317; RRID: AB_493569,BioLegend)
15. Brilliant Violet 421TM anti-mouse I-A/I-E(Clone M5/114.15.2; Cat# 107632; RRID: AB_2650896, BioLegend)
16. Brilliant Violet 605TM-anti-mouse Ly-6C(Clone: HK1.4; Cat#128036; RRID: AB_2562353, BioLegend)
17. PerCP-cyanine5.5-anti-mouse Ly-6G(Clone: 1A8; Cat#127615; RRID: AB_1877272, BioLegend)
18. PE anti-mouse Ly-6G(Clone: 1A8; Cat#127607; RRID: AB_1186104, BioLegend)
19. APC-anti-mouse CD49b (Pan-NK)(Clone: DX5; Cat#108909; RRID: AB_313416, BioLegend)
20. APC anti-mouse IFN-γ(Clone: XMG1.2; Cat#505810; RRID: AB_315404,BioLegend)
21. PE/Cyanine7 anti-mouse TNF-α (Clone: MP6-XT22; Cat#506323; RRID: AB_2204356, BioLegend)
22. FITC- anti-mouse Granzyme B(Clone: GB11; Cat#515403; RRID: AB_2114575,BioLegend)
23.Anti-Mouse CD3 SAFIRE Purified(Clone: 17A2; Cat#05112-25-500, Peprotech)
24. Anti-Mouse CD28 SAFIRE Purified(Clone: 37.51; Cat#10312-25-500, Peprotech)
25. In Vivo Mab anti-mouse CD8α (Clone: 2.43; Cat#BE0061, Bioxcell)
26. Anti-CD8 antibody (Clone: SP16; Cat#ZA-0508, ZSGB-Bio)

Validation

All antibodies used in this study were validated by the supplier as follows:
Trustain FCXTM-anti-mouse CD16/32(https://www.biolegend.com/en-us/products/trustain-fcx-anti-mouse-cd16-32-antibody-5683#productOtherFormats),Brilliant Violet 510TM-anti-mouse CD3(https://www.biolegend.com/en-us/products/brilliant-violet-510-anti-mouse-cd3-antibody-7990),APC anti-mouse CD3(https://www.biolegend.com/en-us/products/apc-anti-mouse-cd3-antibody-8055),Brilliant Violet 421™ anti-mouse CD3ε(https://www.biolegend.com/en-us/products/brilliant-violet-421-anti-mouse-cd3epsilon-antibody-7132),FITC anti-mouse CD3(https://www.biolegend.com/en-us/products/fitc-anti-mouse-cd3-

antibody-45),Alexa 4.Fluor® 700-anti-mouse CD45(https://www.biolegend.com/en-us/products/alexa-fluor-700-anti-mouse-cd45-antibody-3407),FITC-anti-mouse CD4(https://www.biolegend.com/en-us/products/fitc-anti-mouse-cd4-antibody-248),PerCP-cyanine5.5-anti-mouse CD8a(https://www.biolegend.com/en-us/products/percp-cyanine5-5-anti-mouse-cd8a-antibody-4255),PE anti-mouse CD8a(https://www.biolegend.com/en-us/products/pe-anti-mouse-cd8a-antibody-155),FITC anti-mouse CD8a(https://www.biolegend.com/en-us/products/fitc-anti-mouse-cd8a-antibody-153),FITC-anti-mouse CD19(https://www.biolegend.com/en-us/products/fitc-anti-mouse-cd19-antibody-1528),APC-anti-mouseCD11b(https://www.biolegend.com/en-us/products/apc-anti-mouse-human-cd11b-antibody-345),PerCp/Cyanine5.5 anti-mouse/human CD11b(https://www.biolegend.com/en-us/products/percp-cyanine5-5-anti-mouse-human-cd11b-antibody-4257),PE-Cyanine7-anti-mouse CD11c(https://www.biolegend.com/en-us/products/pe-cyanine7-anti-mouse-cd11c-antibody-3086),Brilliant Violet 421TM anti-mouse I-A/I-E(https://www.biolegend.com/en-us/products/brilliant-violet-421-anti-mouse-i-a-i-e-antibody-7147);Brilliant Violet 605TM-anti-mouse Ly-6C(https://www.biolegend.com/en-us/products/brilliant-violet-605-anti-mouse-ly-6c-antibody-8727),PerCP-cyanine5.5-anti-mouse Ly-6G(https://www.biolegend.com/en-us/products/percp-cyanine5-5-anti-mouse-ly-6g-antibody-6116),PE anti-mouse Ly-6G(https://www.biolegend.com/en-us/products/pe-anti-mouse-ly-6g-antibody-4777), APC-anti-mouse CD49b(https://www.biolegend.com/en-us/products/apc-anti-mouse-cd49b-pan-nk-cells-antibody-231),APC anti-mouse IFN-γ(https://www.biolegend.com/en-us/products/apc-anti-mouse-ifn-gamma-antibody-993),PE/Cyanine7 anti-mouse TNF-α(https://www.biolegend.com/en-us/products/pe-cyanine7-anti-mouse-tnf-alpha-antibody-5866),FITC- anti-mouse Granzyme B(https://www.biolegend.com/en-us/products/fitc-anti-human-mouse-granzyme-b-antibody-6066),Anti-Mouse CD3 SAFIRE Purified(https://www.bio-gems.com/anti-mouse-cd3-safire-purified.html),Anti-Mouse CD28 SAFIRE Purified(https://www.bio-gems.com/anti-mouse-cd28-safire-purified.html),In Vivo Mab anti-mouse CD8α(https://bioxcell.com/invivomab-anti-mouse-cd8a-be0061),Anti-CD8 antibody(http://www.zsbio.com/product/ZA-0508).

Eukaryotic cell lines

Policy information about cell lines and Sex and Gender in Research

Cell line source(s)	Murine breast cancer cell lines 4T1 and E0771 were obtained from ATCC.
Authentication	Certified from provider.
Mycoplasma contamination	The cells were tested negative for mycoplasma contamination.
Commonly misidentified lines (See ICLAC register)	No commonly misidentified cell lines were used.

Palaeontology and Archaeology

Specimen provenance	*Provide provenance information for specimens and describe permits that were obtained for the work (including the name of the issuing authority, the date of issue, and any identifying information). Permits should encompass collection and, where applicable, export.*
Specimen deposition	*Indicate where the specimens have been deposited to permit free access by other researchers.*
Dating methods	*If new dates are provided, describe how they were obtained (e.g. collection, storage, sample pretreatment and measurement), where they were obtained (i.e. lab name), the calibration program and the protocol for quality assurance OR state that no new dates are provided.*

☐ Tick this box to confirm that the raw and calibrated dates are available in the paper or in Supplementary Information.

Ethics oversight	*Identify the organization(s) that approved or provided guidance on the study protocol, OR state that no ethical approval or guidance was required and explain why not.*

Note that full information on the approval of the study protocol must also be provided in the manuscript.

Animals and other research organisms

Policy information about studies involving animals; ARRIVE guidelines recommended for reporting animal research, and Sex and Gender in Research

Laboratory animals	Six-week-old female balb/c and C57 mice were purchased from Beijing Vital River Laboratory Animal Technology Co., Ltd (Beijing, China). Mice (4-5 mice per cage) were housed under specific pathogen-free condition with controlled temperature at 22°C-26°C, humidity of 45 ± 5%, and 12 hour light/dark cycle.
Wild animals	No wild animals were used in the study.
Reporting on sex	Female mice. We exclusively utilized female mice in our study, as breast cancer predominantly occurs in women.
Field-collected samples	No field-collected samples were used in the study.

Ethics oversight	The animal experimental procedures were conducted in accordance with the Guidance for the Care of Laboratory Animals and approved by the Ethics Committee of Southern Medical University (Guangzhou, China) or China Pharmaceutical University (Nanjing, China).

Note that full information on the approval of the study protocol must also be provided in the manuscript.

Clinical data

Policy information about clinical studies

All manuscripts should comply with the ICMJEguidelines for publication of clinical research and a completedCONSORT checklist must be included with all submissions.

Clinical trial registration	N/A: not a clinical trial.
Study protocol	N/A: not a clinical trial.
Data collection	As described in Methods. (1) Human stool collection from healthy control individuals and depressed patients was conducted according to the ethical guidelines and approval of the Institutional Review Board of Southeast University Affiliated Zhongda Hospital (Reference 2018ZDSYLL119-P01) . (2) Human blood and breast tissue collection from breast cancer patients with or without depression was conducted according to the ethical guidelines and approval of the Institutional Review Board of Southern Medical University Affiliated Nanfang Hospital (Reference NFEC-2018-038). The depression behavior of patients (including depressed patients and breast cancer patients with depression) was diagnosed with Hamilton depression scale (HAMD-17). Informed consent was obtained from all patients in this study.
Outcomes	1. Fecal gut microbiota compositions of depressed patients and healthy controls (16S rRNA sequencing).<br>2. Fecal contents of small molecule compounds of depressed patients and healthy controls (Metabonomics analysis).<br>3. Serum concentration of acetate in breast cancer patients with or without depression (UPLC-MS/MS).<br>4. The numbers of CD8-positive cells present within the tumors of breast cancer patients with or without depression (Immunohistochemistry).

Dual use research of concern

Policy information about dual use research of concern

Hazards

Could the accidental, deliberate or reckless misuse of agents or technologies generated in the work, or the application of information presented in the manuscript, pose a threat to:

No	Yes	
☐	☐	Public health
☐	☐	National security
☐	☐	Crops and/or livestock
☐	☐	Ecosystems
☐	☐	Any other significant area

Experiments of concern

Does the work involve any of these experiments of concern:

No	Yes	
☐	☐	Demonstrate how to render a vaccine ineffective
☐	☐	Confer resistance to therapeutically useful antibiotics or antiviral agents
☐	☐	Enhance the virulence of a pathogen or render a nonpathogen virulent
☐	☐	Increase transmissibility of a pathogen
☐	☐	Alter the host range of a pathogen
☐	☐	Enable evasion of diagnostic/detection modalities
☐	☐	Enable the weaponization of a biological agent or toxin
☐	☐	Any other potentially harmful combination of experiments and agents

ChIP-seq

Data deposition

☐ Confirm that both raw and final processed data have been deposited in a public database such as GEO.

☐ Confirm that you have deposited or provided access to graph files (e.g. BED files) for the called peaks.

Data access links
May remain private before publication.

For "Initial submission" or "Revised version" documents, provide reviewer access links. For your "Final submission" document, provide a link to the deposited data.

Files in database submission

Provide a list of all files available in the database submission.

Genome browser session
(e.g. UCSC)

Provide a link to an anonymized genome browser session for "Initial submission" and "Revised version" documents only, to enable peer review. Write "no longer applicable" for "Final submission" documents.

Methodology

Replicates

Describe the experimental replicates, specifying number, type and replicate agreement.

Sequencing depth

Describe the sequencing depth for each experiment, providing the total number of reads, uniquely mapped reads, length of reads and whether they were paired- or single-end.

Antibodies

Describe the antibodies used for the ChIP-seq experiments; as applicable, provide supplier name, catalog number, clone name, and lot number.

Peak calling parameters

Specify the command line program and parameters used for read mapping and peak calling, including the ChIP, control and index files used.

Data quality

Describe the methods used to ensure data quality in full detail, including how many peaks are at FDR 5% and above 5-fold enrichment.

Software

Describe the software used to collect and analyze the ChIP-seq data. For custom code that has been deposited into a community repository, provide accession details.

Flow Cytometry

Plots

Confirm that:

☒ The axis labels state the marker and fluorochrome used (e.g. CD4-FITC).

☒ The axis scales are clearly visible. Include numbers along axes only for bottom left plot of group (a 'group' is an analysis of identical markers).

☒ All plots are contour plots with outliers or pseudocolor plots.

☒ A numerical value for number of cells or percentage (with statistics) is provided.

Methodology

Sample preparation

To analyze the immune cells infiltrating the tumor, the tumor tissue was minced on ice and digested in RPMI 1640 medium containing 3mg/ml collagenase II/IV and 10 µg/µl DNase I at 37°C℃ for 60 min. The digestion was stopped by adding RPMI 1640 supplemented with 1% penicillin-streptomycin, 0.5 mM EDTA and 10% FBS. The digested tumor tissue was passed through a 70 µm cell strainer (BD Biosciences) to abtain a single cell suspension. Similarly, the spleen tissue was homogenized by grinding with the plunger of a syringe (2.5 mL) and then filtered through a 70 µm cell strainer. To prevent non-specific binding, the cells were incubated with mouse CD16/CD32 monoclonal antibody (0.25 ug/100 ul) for 15 minutes at room temperature. Subsequently, the cells were stained with appropriate antibodies against s[ecific antigens in a blocking buffer on ice, following the manufacturer's instructions. A viability dye was used to exclude dead cells from the analysis.

Instrument

Flow cytometry was performed on LSR-Fortessa (BD Biosciences) instruments.

Software

FlowJo software (Tree Star).

Cell population abundance

The percentage of targeted cells accounted for the CD45 positive cells were present in the paper.

Gating strategy

The Cells were based on viability and analyzed for various immune cell populations, including CD8+ T cells (CD45+ CD3+ CD8 +), T cells (CD45+ CD3+), B cells (CD45+ CD19+), NK cells (CD45+ CD49b+), macrophages (CD45+ MHCII+ CD11b+ CD11C+), neutrophils (CD45+ CD11b+ Ly6G+), monocytes (CD45+ CD11b+ Ly6C+) , using the FlowJo software (Tree Star).

☒ Tick this box to confirm that a figure exemplifying the gating strategy is provided in the Supplementary Information.

Magnetic resonance imaging

Experimental design

Design type

Indicate task or resting state; event-related or block design.

Design specifications

Specify the number of blocks, trials or experimental units per session and/or subject, and specify the length of each trial or block (if trials are blocked) and interval between trials.

Behavioral performance measures | *State number and/or type of variables recorded (e.g. correct button press, response time) and what statistics were used to establish that the subjects were performing the task as expected (e.g. mean, range, and/or standard deviation across subjects).*

Acquisition

Imaging type(s) | *Specify: functional, structural, diffusion, perfusion.*

Field strength | *Specify in Tesla*

Sequence & imaging parameters | *Specify the pulse sequence type (gradient echo, spin echo, etc.), imaging type (EPI, spiral, etc.), field of view, matrix size, slice thickness, orientation and TE/TR/flip angle.*

Area of acquisition | *State whether a whole brain scan was used OR define the area of acquisition, describing how the region was determined.*

Diffusion MRI ☐ Used ☐ Not used

Preprocessing

Preprocessing software | *Provide detail on software version and revision number and on specific parameters (model/functions, brain extraction, segmentation, smoothing kernel size, etc.).*

Normalization | *If data were normalized/standardized, describe the approach(es): specify linear or non-linear and define image types used for transformation OR indicate that data were not normalized and explain rationale for lack of normalization.*

Normalization template | *Describe the template used for normalization/transformation, specifying subject space or group standardized space (e.g. original Talairach, MNI305, ICBM152) OR indicate that the data were not normalized.*

Noise and artifact removal | *Describe your procedure(s) for artifact and structured noise removal, specifying motion parameters, tissue signals and physiological signals (heart rate, respiration).*

Volume censoring | *Define your software and/or method and criteria for volume censoring, and state the extent of such censoring.*

Statistical modeling & inference

Model type and settings | *Specify type (mass univariate, multivariate, RSA, predictive, etc.) and describe essential details of the model at the first and second levels (e.g. fixed, random or mixed effects; drift or auto-correlation).*

Effect(s) tested | *Define precise effect in terms of the task or stimulus conditions instead of psychological concepts and indicate whether ANOVA or factorial designs were used.*

Specify type of analysis: ☐ Whole brain ☐ ROI-based ☐ Both

Statistic type for inference (See Eklund et al. 2016) | *Specify voxel-wise or cluster-wise and report all relevant parameters for cluster-wise methods.*

Correction | *Describe the type of correction and how it is obtained for multiple comparisons (e.g. FWE, FDR, permutation or Monte Carlo).*

Models & analysis

n/a	Involved in the study
☐	☐ Functional and/or effective connectivity
☐	☐ Graph analysis
☐	☐ Multivariate modeling or predictive analysis

Functional and/or effective connectivity | *Report the measures of dependence used and the model details (e.g. Pearson correlation, partial correlation, mutual information).*

Graph analysis | *Report the dependent variable and connectivity measure, specifying weighted graph or binarized graph, subject- or group-level, and the global and/or node summaries used (e.g. clustering coefficient, efficiency, etc.).*

Multivariate modeling and predictive analysis | *Specify independent variables, features extraction and dimension reduction, model, training and evaluation metrics.*
